# The crystal structure of the *N*-acetylglucosamine 2-epimerase from *Nostoc* sp. KVJ10 reveals the true dimer

**DOI:** 10.1107/S2059798318017047

**Published:** 2019-01-08

**Authors:** Marie-Josée Haglund Halsør, Ulli Rothweiler, Bjørn Altermark, Inger Lin Uttakleiv Raeder

**Affiliations:** a The Norwegian Structural Biology Centre (NorStruct), Department of Chemistry, UiT – The Arctic University of Norway, 9037 Tromsø, Norway

**Keywords:** *N*-acetylglucosamine 2-epimerase, AGE, sialic acid, crystal packing, ManNAc, GlcNAc, *N*-acetylmannosamine, *Nostoc* sp. KVJ10

## Abstract

*N*-Acetylglucosamine 2-epimerases (AGEs) catalyze the interconversion of *N*-acetylglucosamine and *N*-acetylmannosamine. They can be used to perform the first step in the synthesis of sialic acid from *N*-acetylglucosamine, which makes the need for efficient AGEs a priority. This study presents the structure of the AGE from *Nostoc* sp. KVJ10 collected in northern Norway, referred to as nAGE10. It is the third AGE structure to be published to date, and the first one in space group *P*4_2_2_1_2. The nAGE10 monomer folds as an (α/α)_6_ barrel in a similar manner to that of the previously published AGEs, but the crystal did not contain the dimers that have previously been reported. The previously proposed ‘back-to-back’ assembly involved the face of the AGE monomer where the barrel helices are connected by small loops. Instead, a ‘front-to-front’ dimer was found in nAGE10 involving the long loops that connect the barrel helices at this end. This assembly is also present in the other AGE structures, but was attributed to crystal packing, even though the ‘front’ interface areas are larger and are more conserved than the ‘back’ interface areas. In addition, the front-to-front association allows a better explanation of the previously reported observations considering surface cysteines. Together, these results indicate that the ‘front-to-front’ dimer is the most probable biological assembly for AGEs.

## Abstract 2

The *N*-acetylglucosamine 2-epimerase (AGE) from *Nostoc* sp. KVJ10 (nAGE10) was crystallized in a different space group to other AGEs. The nAGE10 dimer, while different from the proposed AGE dimers in previously published structures, can also be found in these structures and is probably the biological dimer.

## 1. Introduction   

*N*-Acetylglucosamine 2-epimerases (AGEs; EC 5.1.3.8) catalyze the reversible epimerization of *N*-acetylmannosamine (ManNAc) to *N*-acetylglucosamine (GlcNAc) in both eukary­otic and prokaryotic cells (Ghosh & Roseman, 1965 ▸; Lee, Chien *et al.*, 2007 ▸). It seems that their role, at least in mammals, is to divert the metabolic flux away from sialic acid synthesis (Luchansky *et al.*, 2003 ▸). The reaction follows a deprotonation/reprotonation mechanism involving two key residues, which had been thought to be histidines, but which have recently been identified as a glutamine and an arginine (Lee, Wu *et al.*, 2007 ▸; Takahashi, Takahashi *et al.*, 2001 ▸; Wang *et al.*, 2016 ▸). AGE activity is greatly enhanced by the presence of nucleotides, in particular ATP, which serve as allosteric activators (Datta, 1970 ▸; Ghosh & Roseman, 1965 ▸; Tabata *et al.*, 2002 ▸; Takahashi, Hori *et al.*, 2001 ▸).

From an industrial point of view, AGEs can be used for the synthesis of *N*-acetylneuraminic acid (Neu5Ac), which is also known as sialic acid (Hu *et al.*, 2010 ▸; Kragl *et al.*, 1991 ▸; Lee *et al.*, 2004 ▸; Lee, Chien *et al.*, 2007 ▸; Maru *et al.*, 1996 ▸; Tabata *et al.*, 2002 ▸; Wang *et al.*, 2009 ▸). One current approach is a one-pot, coupled reaction with an *N*-acetylneuraminic acid lyase (NAL; EC 4.1.3.3), in which the AGE performs the (reverse) GlcNAc-to-ManNAc epimerization and the NAL performs the condensation with pyruvate, thus producing Neu5Ac. However, there are several challenges related to the use of AGEs in this step, such as the unfavourable thermodynamic equilibrium, the requirement for ATP and the inhibition by both pyruvate and Neu5Ac (Datta, 1970 ▸; Ghosh & Roseman, 1965 ▸; Klermund *et al.*, 2013 ▸; Kragl *et al.*, 1991 ▸).

In order to optimize Neu5Ac production, the search for better AGEs is a current area of focus and enzymes from cyanobacterial sources seem to be the most promising. Indeed, the AGEs from *Anabaena* sp. CH1 (AnaAGE) and *A. variabilis* ATCC 29413 (AvaAGE) have a specific activity that is almost four times that of the porcine enzyme, despite having similar affinities for GlcNAc (Klermund *et al.*, 2013 ▸; Lee, Wu *et al.*, 2007 ▸). Other enzymes present interesting properties, such as the AGE from *Bacteroides ovatus* ATCC 8483 (BoAGE), which shows a much higher affinity for GlcNAc than ManNAc, and that from *Synechocystis* PCC 6803 (SynAGE), which has the lowest *K*_m_ values for both GlcNAc and ManNAc of all AGEs that have been characterized to date (Sola-Carvajal *et al.*, 2012 ▸; Tabata *et al.*, 2002 ▸).

In addition to bioprospecting for enzymes that are more suited for industrial purposes, the identification of the structural elements that govern the activity parameters of AGEs is a priority. Two AGEs had been crystallized prior to this study: the porcine enzyme (pAGE; PDB entry 1fp3) and that from *Anabaena* sp. CH1 (AnaAGE; PDB entry 2gz6), the latter of which used the former as a template for molecular replacement (Itoh *et al.*, 2000 ▸; Lee, Wu *et al.*, 2007 ▸). These studies revealed that the overall structure of AGEs is that of a (α/α)_6_ barrel. Together with mutagenesis studies, this led to the proposal of a reaction mechanism involving two critical histidine residues as acid/base catalysts in the protonation/deprotonation of carbon C2 of GlcNAc/ManNAc. The enzyme from *Pedobacter heparinus* DSM2366 (PhGn2E) was used in a hydrogen/deuterium-exchange experiment, which confirmed this type of mechanism for the epimerization, albeit with a glutamine/arginine pair as catalysts (Wang *et al.*, 2016 ▸).

AGEs were found to be structurally similar to sulfoquinovose isomerases encoded by the *yihS* gene (EC 5.3.1.31) and to cellobiose 2-epimerases (CE; EC 5.1.3.11), despite a relatively low sequence identity (Denger *et al.*, 2014 ▸; Fujiwara *et al.*, 2013 ▸, 2014 ▸; Itoh *et al.*, 2008 ▸; Tyler & Leatherwood, 1967 ▸). Together, they form the AGE superfamily (Pfam accession No. PF07221), for which 13 structures are currently available (Bateman *et al.*, 2004 ▸; Finn *et al.*, 2014 ▸). Several of these structures have been solved as protein–ligand complexes, and structural comparison of the relatively conserved active sites helped to form the current hypothesis for the catalytic mechanism of AGEs (Itoh *et al.*, 2008 ▸).

In this study, we report the determination of the crystal structure of the AGE from the local strain *Nostoc* sp. KVJ10, which we refer to as nAGE10 (Liaimer *et al.*, 2016 ▸). We also demonstrate that the most probable biological assembly for the AGE dimer involves the opposite face of the barrel to that published previously. Finally, we reveal the presence of a putatively conserved chloride ion within the active site of AGE.

## 2. Materials and methods   

### 2.1. Cloning, expression and purification   

The gene coding for nAGE10, containing the coding sequence for a C-terminal hexahistidine tag and optimized for expression in *Escherichia coli*, was synthesized by GeneArt and subcloned into the pDEST14 expression vector using the Gateway cloning system (ThermoFisher Scientific). The protein was expressed in *E. coli* BL21 Star (DE3) cells (ThermoFisher Scientific), which were grown at 37°C to an OD_600_ of 0.6, brought to 20°C and induced with 0.5 m*M* isopropyl β-d-1-thiogalactopyranoside (IPTG; Sigma–Aldrich). The cells were incubated for approximately 16 h at 20°C and harvested by centrifugation at 6000*g* for 25 min. Bacterial cell pellets were resuspended in 50 m*M* Tris–HCl pH 7.5, 250 m*M* NaCl, 5 m*M* β-mercaptoethanol, 10 m*M* imidazole, along with half a tablet of protease-inhibitor cocktail (Roche) and 2 µl DNAse I. The cells were disrupted using a French press and the extract was centrifuged at 20 000*g* for 2 × 30 min to remove cell debris. The supernatant was filtered and purified by affinity chromatography using a 5 ml HisTrap column (GE Healthcare). The binding buffer consisted of 20 m*M* sodium phosphate pH 7.4, 0.5 *M* NaCl, 20 m*M* imidazole. The elution buffer consisted of 20 m*M* sodium phosphate pH 7.4, 0.5 *M* NaCl, 500 m*M* imidazole. The protein was eluted using a gradient from 5% to 80% elution buffer over 60 ml. After purification, fractions containing protein were assessed by SDS–PAGE. Pure fractions were pooled and dialyzed overnight in 50 m*M* Tris–HCl pH 7.5, 250 m*M* NaCl. The protein solutions were further dialyzed into storage buffer (20 m*M* HEPES pH 7.4, 115 m*M* NaCl). The oligomerization state of nAGE10 was determined by gel-filtration chromatography on a Superdex 75 10/300 GL column (GE Healthcare). The results are presented in the supporting information. The protein concentration was determined by measuring the absorbance at 280 nm using a theoretical extinction coefficient for the protein.

### 2.2. Differential scanning calorimetry   

Differential scanning calorimetry (DSC) was used to determine the melting temperature of nAGE10. Thermal denaturation of nAGE10 was followed between 5 and 95°C using a heating/cooling rate of 1°C min^−1^. A solution of nAGE10 at 1.5 mg ml^−1^ in storage buffer was used in the experiment. The results are presented in the supporting information.

### 2.3. Structure determination   

The protein was concentrated to 10 mg ml^−1^ in storage buffer prior to crystallization. Screening for crystallization conditions was performed using the sitting-drop method (drop size of 200 + 200 nl). The initial screening was performed using in-house and commercially available screens at both room temperature and 4°C. From this, two initial hits were obtained (at both temperatures) with 0.1 *M* sodium acetate pH 5, 17.6% PEG 6000 or with a combination of 0.07 *M* sodium acetate pH 5, 0.05 *M* calcium acetate and 12% PEG 3350. The optimized crystallization condition was 0.1 *M* sodium acetate pH 5, 0.1 *M* calcium acetate, 10% PEG 3350, 3% dextran. Crystals were obtained at room temperature after 1–3 days of incubation and were flash-cooled in liquid nitrogen. 20% ethylene glycol was used as a cryoprotectant. Crystallographic data were collected at the BESSY II photon source, Helmholtz-Zentrum Berlin, Germany. The images were integrated using *XDSapp* (Sparta *et al.*, 2016 ▸). The structure was solved by molecular replacement using the AnaAGE structure (PDB entry 2gz6) as a search model (Lee, Wu *et al.*, 2007 ▸). Refinement was performed using *PHENIX* and the *CCP*4 program *REFMAC*5 (Adams *et al.*, 2010 ▸; Murshudov *et al.*, 2011 ▸; Winn *et al.*, 2011 ▸). The waters were placed by *Coot* v.0.7.2 (Emsley *et al.*, 2010 ▸). The crystallographic data and model statistics are summarized in Table 1 ▸.

### 2.4. Analysis of interface areas   

The surfaces of nAGE10, AnaAGE and pAGE were analyzed using the *PDBePISA* server in order to obtain parameters pertaining to the structural and chemical properties of possible assemblies (Krissinel & Henrick, 2007 ▸).

### 2.5. Sequence comparison   

The amino-acid sequence of nAGE10 and the previously characterized AGEs were aligned using the *MUSCLE* multiple sequence alignment tool with default settings (Edgar, 2004*a* ▸,*b* ▸). Secondary-structure data for nAGE10 and pAGE were obtained from their respective PDB files. The graphical output was created using the *TeXshade* package for LaTeX (Beitz, 2000 ▸).

### 2.6. Enzyme-activity measurement   

The activity of nAGE10 was assessed by coupling the epimerization to the condensation reaction catalyzed by the NAL from *Aliivibrio salmonicida* (AsNAL; EC 4.1.3.3; PDB entry 5afd; M. K. Gurung, B. Altermark, I. L. U. Rader, R. Helland & A. O. Smalas, unpublished work), resulting in the production of Neu5Ac. Its presence was detected using the thiobarbituric acid (TBA) assay (Warren, 1959 ▸). Samples consisting of 124 m*M* HEPES pH 8.0, either 15 m*M* GlcNAc or 15 m*M* ManNAc, 10 m*M* ATP, 15 m*M* pyruvate, 7 µg NAL and 10 µg AGE10 were incubated at room temperature for 1 h. The reactions were terminated by adding 137 µl sodium periodate (2.5 mg ml^−1^ sodium periodate in 57 m*M* H_2_SO_4_) followed by incubation at 37°C for 15 min with shaking. Arsenite solution (50 µl, 25 mg ml^−1^ in 0.5 *M* HCl) was added, resulting in a brown colour. The tubes were shaken until the brown colour disappeared and 100 µl TBA solution (71 mg ml^−1^ TBA pH 9.0) was then added. The samples were placed in boiling water for 7.5 min and then on ice for 5 min; they were then brought to room temperature for 5 min. Acidic *n*-butanol (5% HCl, 1 ml) was added and the samples were shaken for 10 min at room temperature. The tubes were spun down at 16 060*g* for 7 min and the absorbance of the upper layer was measured at 549 nm on a SpectraMax (Molecular Devices). A molar extinction coefficient of 57 000 *M* ^−1^ cm^−1^ was used for concentration calculations (Warren, 1959 ▸).

### 2.7. Graphical output generation   

Molecular representations were generated using *PyMOL* (v.1.8; Schrödinger) and sequence alignments were rendered with the *TeXshade* package for LaTeX (Beitz, 2000 ▸). Figure editing was performed in *Inkscape* (https://inkscape.org/).

## 3. Results and discussion   

### 3.1. Structure of nAGE10   

nAGE10 was crystallized using the hanging-drop vapour-diffusion method. Just as for pAGE (PDB entry 1fp3) and AnaAGE (PDB entry 2gz6), nAGE10 crystallized at a pH below 6. Attempts to either crystallize it at a higher pH or to increase the pH of the existing crystals were unsuccessful. Co-crystallization trials with GlcNAc, ManNAc or ATP, as well as a combination of the latter with hexosamine, did not result in crystallized complexes, although crystals of the apoenzyme were obtained from these experiments. Soaking did not affect the crystals. These results are similar to those described for previously published AGE structures, indicating that this behaviour may be inherent to AGEs rather than to the crystallization method. Temperature did not seem to play a significant role in the crystallization process, as similar crystals were obtained at both room temperature and 4°C.

The nAGE10 crystal diffracted to 1.70 Å resolution, and the subsequent model, which was determined by molecular replacement using the AnaAGE structure (PDB entry 2gz6; 90% sequence identity), was refined to an *R* factor of 0.179 (*R*_free_ = 0.209). The r.m.s.d. between the two structures is 0.8 (Holm & Laakso, 2016 ▸). The refinement data are summarized in Table 1 ▸. In contrast to the pAGE and AnaAGE crystals, which both belonged to space group *P*2_1_2_1_2_1_, the nAGE10 crystal belonged to space group *P*4_2_2_1_2.

**Figure 1:**
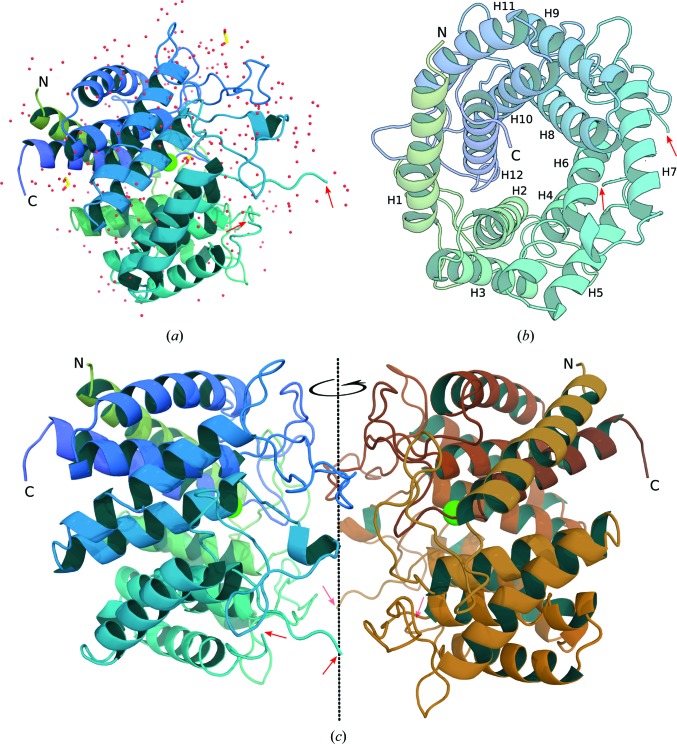
Structural overview of nAGE10. (*a*) The contents of the asymmetric unit of the nAGE10 structure, with the nAGE10 monomer shown from the side in a cartoon representation. It is coloured using a green–blue palette from the N-terminal to the C-terminal residues. Waters are shown as red, nonbonded spheres. Ethylene glycol molecules (three) are represented as yellow sticks and the buried chloride ion as a green sphere. (*b*) The nAGE10 monomer, shown from the front (the face where the α-helices that form the barrel are linked by short loops). Each helix is numbered (H1–H12) according to its placement in the amino-acid sequence. (*c*) The nAGE10 dimer. Generation of symmetry mates within 3 Å of nAGE10 reveals the presence of an additional monomer for which dimer association is probable involving the fronts of the monomers. Red arrows indicate the extremities of the disordered loop (residues 156–165).

The asymmetric unit contained one nAGE10 monomer, a chloride ion and three ethylene glycol molecules, as well as 318 waters (Fig. 1 ▸ *a*). The structure of the monomer was resolved for the amino-acid sequence from Tyr3 to Leu391, with residues 157–164 missing. The fold is that of an (α/α)_6_ barrel, with the helices connected by small loops at one end (the back) and long loops at the other end (the front). The nAGE10 barrel is composed of two concentric rings of six helices each, in which the sequence orientation is opposite. Helices H1, H3, H5, H7, H9 and H11 form the outer ring of the barrel, with the even-numbered helices as the inner ring (Fig. 1 ▸ *b*). The nAGE10 barrel is about 50 Å in diameter and 30 Å in length.

### 3.2. Dimer assembly   

Fig. 1 ▸(*c*) shows nAGE10 as a dimer, which was generated by twofold symmetry using the operation *y* − 1, *x* + 1, −*z*. The calculated interaction interface represents 9.1% of the accessible surface area, but this value is most likely to be underestimated owing to the disordered loop between residues 156 and 165 (indicated by red arrows in Fig. 1 ▸ *c*). The interface is composed of 41 residues for each monomer, 35 of which participate in extensive interactions (Table 2 ▸). 11 hydrogen bonds involve the residue pairs Asp48/Arg234, Asp50/Arg167, Pro108/Thr166 (which only occurs once), Gln295/Lys367, Leu297/Gln301 and Trp299/Leu360. In addition, 53 pairs of residues form van der Waals (vdW) interactions. The proposed interactions involving residues Thr166 and Arg167 are probably influenced by the disorder of the aforementioned loop.

**Figure 2:**
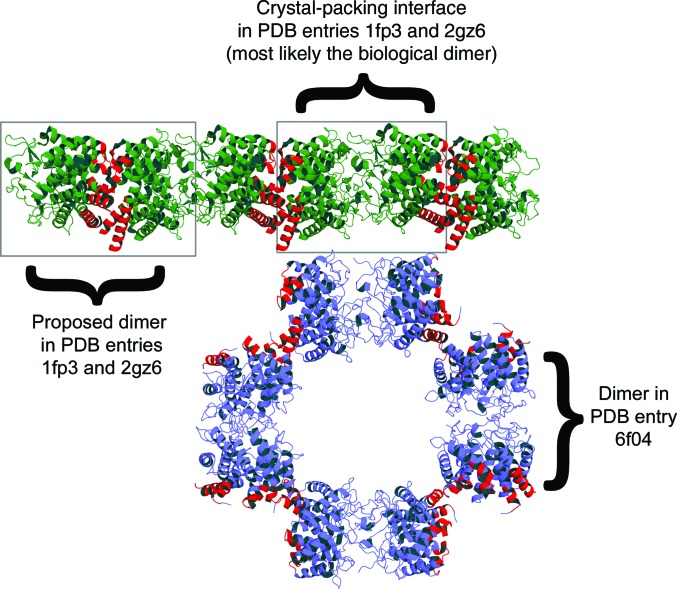
Comparison of the nAGE10 and pAGE dimers. Parts of the unit cell for pAGE (PDB entry 1fp3; green) and nAGE10 (PDB entry 6f04; blue) are shown to illustrate the different crystal packings of these structures. The residue ranges that are involved in the back-to-back interface of PDB entry 1fp3 and the corresponding ranges in nAGE10 are coloured red. PDB entry 2gz6 (AnaAGE) has the same crystal packing as PDB entry 1fp3 and was therefore omitted to avoid redundancy.

The nAGE10 dimer, which involves the front faces of each monomer, differs from the back-to-back association presented for AnaAGE and pAGE (Itoh *et al.*, 2000 ▸; Lee, Wu *et al.*, 2007 ▸). A front-to-front association for AnaAGE (symmetry operator *x* − 1, *y*, *z*) is mentioned by Lee *et al.* (2007 ▸) and is attributed to crystal packing. However, the nAGE10 dimer can be superimposed onto that of AnaAGE assuming that the front-to-front packing in PDB entry 2gz6 is the biological dimer (instead of the back-to-back packing). pAGE, which has the same crystal packing as AnaAGE, also has a front-to-front dimer (Fig. 2 ▸). A comparison of the unit cell of nAGE10 with those of pAGE and AnaAGE reveals not only that the back-to-back assembly does not exist in the nAGE10 crystal, but also that either the front-to-front or the back-to-back assemblies can be used as the asymmetric unit for the pAGE and AnaAGE structures, as presented in Fig. 2 ▸.

A comparison of the assembly parameters calculated by the *PISA* method indicates that the front-to-front organization is more favourable than the back-to-back organization in terms of interface area, number of interactions and solvation energy (Krissinel & Henrick, 2007 ▸). For AnaAGE, the interface area is only 346.8 Å^2^ for the back-to-back assembly, while it is 1386.3 Å^2^ for the front-to-front complex (again, the missing residues may be part of the dimer interface, so the interface area for the front-to-front assembly is probably larger). The two interfaces of pAGE have similar areas (1227.2 Å^2^ for the front and 1029 Å^2^ for the back), but the solvation energy is much more favourable for the front-to-front assembly at −22.3 kcal mol^−1^, compared with −1.2 kcal mol^−1^ for the back-to-back assembly. In terms of interactions, Itoh *et al.* (2000 ▸) reported that nine hydrogen bonds and 23 vdW contacts (<4.5 Å) occur between monomers in the back-to-back assembly. Using the same criteria, 13 hydrogen bonds and 52 vdW contacts can be found for the front-to-front dimer (Table 2 ▸). In the case of AnaAGE the front-to-front assembly is mentioned (as crystal packing), but only the interactions involving eight residues are described. Using the same criteria as the authors for identifying interactions and considering only hydrogen bonds leads to the involvement of 14 residues (Table 2 ▸). For example, the residues involved in the polar interactions that take place between Asp49 and Arg166, between Pro107 and Thr165 and between Trp298 and Leu359 below 3.5 Å are described, but not those between Asp47 and Arg233, between Leu296 and Gln300 and between Arg355 and Glu357 that also occur within the same distance (data not shown).

**Figure 3:**
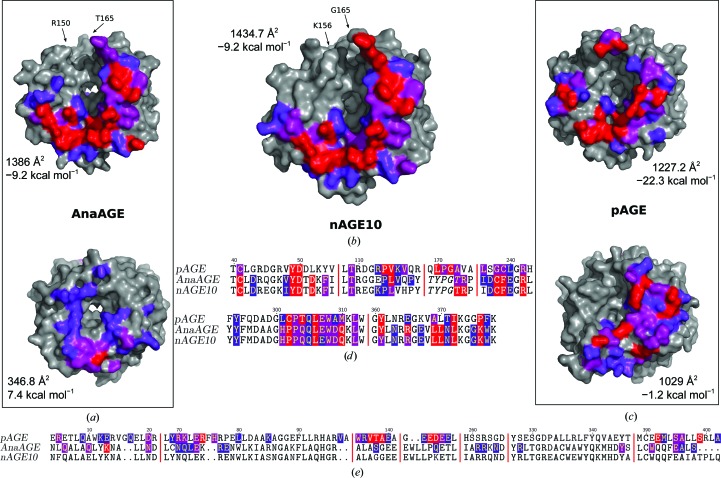
Dimer-interaction interfaces of AGEs as a function of buried area. The AGE monomers are represented as a surface and are coloured grey. Residues involved in dimer interactions, as calculated by *PISA*, are coloured purple (less than 30% buried), pink (less than 60% buried) and red (over 60% buried). The residues at each side of the missing loop are indicated by arrows and labelled. (*a*) AnaAGE. Top, front; bottom, back. (*b*) nAGE10 (front). (*c*) pAGE. Top, front; bottom, back. (*d*, *e*) Sequence alignment showing the interface residues using the same colour scheme as used for the surface representations. (*d*) Front. Residues which are unresolved in the crystal structures are shown in italics. (*e*) Back.

A look at the distribution of the interface residues, which is presented in Fig. 3 ▸, shows that the back interface areas of AnaAGE and pAGE are quite different (bottom surfaces in Figs. 3 ▸ *a* and 3 ▸ *c*). This is explained by the fact that the residues involved in this interface are not conserved between AnaAGE and pAGE (Fig. 3 ▸ *e*). Indeed, the main interaction patch of pAGE, which involves the loops between H4 and H5 (residues 135–140) and between H6 and H7 (residues 194–198), is missing in AnaAGE. The shape of these loops is thus different and the monomers are further apart at this location in AnaAGE than they are in pAGE (data not shown). On the contrary, the residues involved in the front interface area are mostly conserved, with the exception of the loop between H5 and H6 (residues 170–173; Fig. 3 ▸ *d*). This loop is also only resolved for pAGE, and it is likely that the interface parameters for the cyanobacterial AGEs, in particular the solvation energy, may be lower than calculated.

In addition to this, it was shown that cysteines are involved in the dimerization of pAGE (Takahashi *et al.*, 1988 ▸). The front interface contains three cysteines (residues 41, 239 and 302 of pAGE), which is the exact number of cysteines that were alkylated by the treatment with *N*-ethylmaleimide performed in that study. Those cysteines lose at least 55% of their solvent-accessible area upon dimer formation, with Cys239 and Cys302 involved in hydrogen bonds to the neighbouring monomer (Table 2 ▸). Their positions are identical to those in human AGE when mapping the hAGE sequence onto the pAGE structure (data not shown); mutation studies revealed the importance of Cys41 for the stability of hAGE, while Cys239 and Cys302 do not seem to play critical roles (Takahashi, Takahashi *et al.*, 2001 ▸; Takahashi, Takahashi, Kaneko, Ogasawara, Shindo, Saito *et al.*, 1999 ▸).

### 3.3. Dimer organization and ATP-binding site   

One of the principal consequences of the front-to-front dimer is for the ATP-binding site. To date, two hypotheses have been put forward regarding the location of this site. One involves the H5/H6 loop and the other involves a glycine-rich fragment (residues 363–369 of AnaAGE) in the C-terminus (Lee, Chien *et al.*, 2007 ▸; Liao *et al.*, 2012 ▸; Sola-Carvajal *et al.*, 2012 ▸; Takahashi *et al.*, 2002 ▸, 2005 ▸). While the latter, which is based on sequence similarity to the motif of NTPases, is conserved across species, the evidence supporting the non­conserved H5/H6 loop is built on experiments using chimeric constructs, mutagenesis and ATP footprinting. A nonconserved binding region would also better explain the differences in nucleotide affinities that are observed across both species and nucleotide type (Lee, Chien *et al.*, 2007 ▸; Sola-Carvajal *et al.*, 2012 ▸; Takahashi, Hori *et al.*, 2001 ▸). This would mean that the H5/H6 loop is involved in both dimer inter­action and ATP binding. However, the second putative site corresponds to part of the H11/H12 loop, which is also on the front face of the monomer in a position opposite to that of the H5/H6 loop. The loop points towards the centre of the barrel, and if the H5/H6 loop does the same, which is the case for the corresponding loop in pAGE, they might be in proximity to each other. This opens the possibility of both proposed sites being involved. Independently of which site is used, the localization of the ATP-binding site at the dimer interface is the determinant of its role in AGE activity, which may involve the oligomeric state of the enzyme. The inability to obtain crystals of the AGE–ATP complex either by co-crystallization or soaking could indicate the dissociation of the dimeric form upon ATP binding, as has been seen in different complexes (Nayar & Bhattacharyya, 1997 ▸; Ahmed *et al.*, 2015 ▸; Du *et al.*, 2014 ▸).

### 3.4. Active site and comparison with the AGE superfamily   

**Figure 4:**
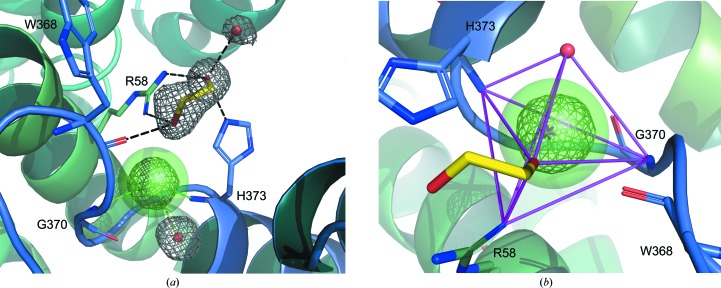
Structure of the active site of nAGE10. nAGE10 is shown in a cartoon representation and is coloured in a green–blue palette from the N-terminus to the C-terminus. Ethylene glycol (yellow) and active-site residues are shown as sticks, and waters and the chloride ion are shown as nonbonded spheres. (*a*) Active site. Electron density (2*F*_o_ − *F*_c_ map) is shown around the waters, the chloride ion and the ethylene glycol molecule. Polar contacts for ethylene glycol are coloured black and coordination of the chloride ion is shown in green. (*b*) Coordination geometry for the buried chloride ion. The five coordinating atoms form a trigonal bipyramid (magenta) with the chloride at the centre of mass (magenta star).

The active site of nAGE10, which is presented in Fig. 4 ▸(*a*), contains an ethylene glycol molecule and a buried chloride ion. No ligand could be identified, although the enzyme was crystallized in the presence of ManNAc. The ethylene glycol interacts with the side chains of His373 and Arg58, as well as the main chain of Trp368 and a water molecule. The chloride ion is coordinated by the amine groups of His373 and the Gly370 main chain, which are within its first coordination sphere (3.4 Å), along with a water molecule (Carugo, 2014 ▸). However, the geometry of this three-atom coordination seems to be incorrect, as the chloride is completely outside the plane formed by these atoms. By investigating nearby atoms, the best geometry was obtained for a five-atom coordination, with the ethylene glycol (3.7 Å) and Arg58 (3.5 Å) as the two remaining partners (Fig. 4 ▸ *b*). Other potential partners are the amine group of Phe372, the Gly370 carbonyl and the δ position of His373. The latter would mean that His373 is flipped, which has consequences for the interactions taking place within the active site.

The structures of pAGE and AnaAGE contain only waters within their active sites, and the active-site residues are conserved compared with those of nAGE10 (not shown). However, it is worth noting that for each of them there is a water molecule at the position where the chloride ion is found in the nAGE10 structure (water 389 in AnaAGE and water 521 in pAGE). The closest neighbour to both molecules is at 3.1 Å, and their *B* factors are below 3 Å^2^, while those of the neighbouring water molecules range from 11.9 to 29.3 Å^2^.

**Figure 5:**
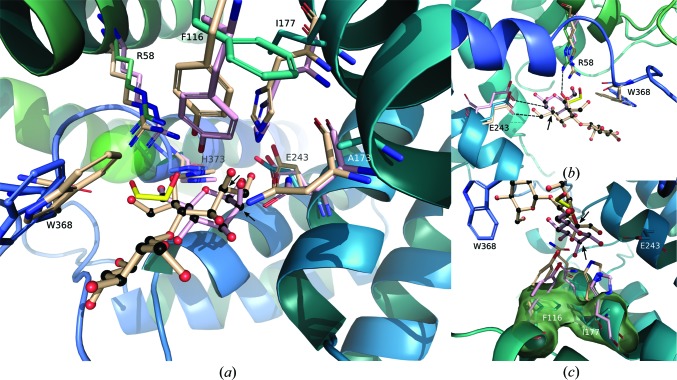
Comparison of active sites in the AGE family. The active sites of RmCE in complex with cellobiitol (wheat; PDB entry 3wki) and SeYihS in complex with mannose (light pink; PDB entry 2zbl) are superimposed onto the structure of nAGE10 (blues, with ethylene glycol in yellow). The C2 atoms of the mannose rings of cellobiitol and mannose are indicated by black arrows. Residues of nAGE10 are labelled. (*a*) Overview. Perspective was added for visualization purposes. (*b*) Detail of the catalytic residues and the interaction with Trp368. (*c*) Detail of the *N*-acetyl group binding pocket.

While none of the AGEs could be co-crystallized with ligands, several protein–ligand complex structures are available for other members of the AGE superfamily. A superimposition of the active sites of the cellobiose epimerase from *Rhodothermus marinus* (RmCE; PDB entry 3wki) and the isomerase YihS from *Salmonella enterica* (SeYihS; PDB entry 2zbl) is presented in Fig. 5 ▸ (Fujiwara *et al.*, 2014 ▸; Itoh *et al.*, 2008 ▸). It shows that the ethylene glycol molecule in the active site of nAGE10 is in proximity to the O5, C5, C6 and O6 positions of mannose, as well as the corresponding positions of cellobiitol. This placement is consistent with the suggested role of Arg63 of the epimerase PhGn2E as the agent that is responsible for the protonation and deprotonation of ManNAc (Wang *et al.*, 2016 ▸). Indeed, the corresponding position in AGE10 (Ar68) is only 2.9 Å from the oxygen of ethylene glycol that mimicks O5 of mannose (Fig. 5 ▸ *b*). The other catalytic residue, Glu314 in PhGn2E (Glu309 in nAGE10), is surprisingly not in the vicinity of the ligands, with those in SeYihS (Gln320) and RmCE (Gln326) being more than 7 Å away from the C2 atom of their respective ‘mannoses’ (data not shown). Glu251 in SeYihS (Glu243 in nAGE10) is much closer: it is only 4.4 Å from the C2 atom.

Another interesting feature is the stacking interaction between Trp385 of RmCE and the glucose ring of cellobiitol (the same interaction is present with epilactose and glucosyl­mannose in the other RmCE structures; PDB entries 3wkg and 3wkh). This residue, which corresponds to Trp368 in nAGE10, is strictly conserved in AGEs but its function has not yet been investigated. Considering the position of the glucose relative to the mannose ring, it may be involved in the substrate-specificity differences observed by Wang *et al.* (2016 ▸) when using derivatives of glucosamine substituted at C4 (just like the glucose ring of cellobiitol is linked to C4 of the open mannose ring).

A structural comparison of the active sites within the AGE superfamily also reveals a putative binding site for the *N*-acetyl group of GlcNAc/ManNAc in AGEs. For RmCE (and SeYihS), the C2 position of the ‘mannose’ and the OH that it carries interact with the side chains of Tyr124 (Tyr111), His200 (His176) and Asn196 (Asn172). Those positions are not conserved in nAGE10, where they are replaced by Phe116, Ile177 and Ala173, respectively (Fig. 5 ▸ *c*). This leaves a cavity that would be large enough to accommodate an *N*-acetyl group and would also explain why PhGn2E is not active on glucos­amine, as it would not be retained in the active site. All three residues are either almost or strictly conserved in AGEs.

RmCE also possesses a chloride ion within its active site, at the same location as that in nAGE10, and the coordination by ethylene glycol seen in nAGE10 may not reflect what takes place in the presence of the substrate. The structure of another CE from *Ruminococcus albus* (RaCE; PDB entry 3vw5; Fujiwara *et al.*, 2013 ▸) contains a water (water 451) at the ‘chloride location’, with a similar *B* factor to those of the neighbouring waters. Investigating the residues corresponding to coordinating residues in RmCE reveals that Gly387 is replaced by a cysteine (Cys371) in RaCE. In *yihS*-encoded proteins the spatial organization of waters differs from those in CEs and AGEs, despite their shared fold (PDB entries 2zbl, 2afa and 2rgk; Itoh *et al.*, 2008 ▸; SGX Research Center for Structural Genomics, unpublished work). The coordinating glycine is replaced in this case by an aspartate (Asp380 in SeYihS) that is conserved within the structures. This strengthens the hypothesis of a conserved chloride within the AGE active site and opens the possibility of studying its role by mutating the glycine position of AGEs.

### 3.5. Sequence similarity to characterized AGEs   

**Figure 6:**
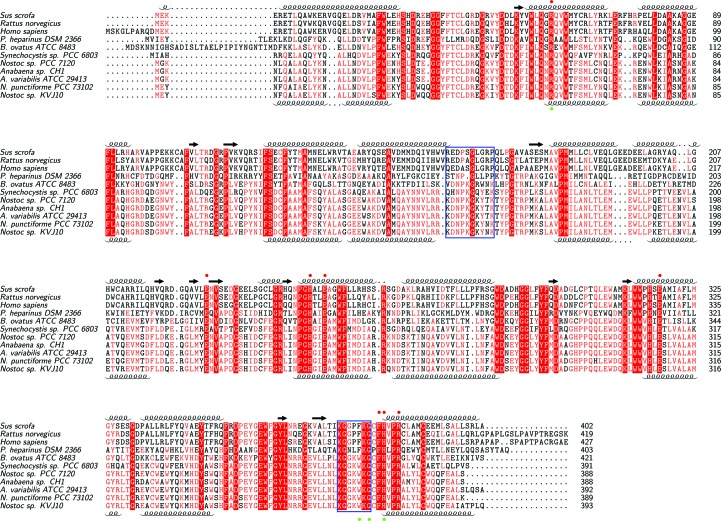
Sequence conservation amongst AGEs. The sequences (with accession numbers in parentheses) are from *Sus scrofa* (NP_999065.1), *Rattus norvegicus* (NP_112357.1), *Homo sapiens* (NP_002901.2), *P. heparinus* DSM2366 (ACU05446.1), *B. ovatus* ATCC 8483 (EDO12673.1), *Synechocystis* sp. PCC 6803 (BAA18210.1), *Nostoc* sp. PCC 7120 (WP_010997838.1), *Anabaena* sp. CH1 (ABG57043.1), *A. variabilis* ATCC 29413 (WP_011320279.1), *N. punctiforme* PCC 73101 (WP_012409471.1) and *Nostoc* sp. KVJ10 (NNBT01000060.1). Identical residues are shown in white on a red background and similar residues in red. α-Helices and β-strands correspond to those in the structures of the AGEs from pig (top; PDB entry 1fp3) and *Nostoc* sp. KVJ10 (bottom). Red circles (top) indicate residues that participate in catalysis in the AGEs from *P. heparinus* DSM 2366 and *Anabaena* sp. CH1 (Lee, Wu *et al.*, 2007 ▸; Wang *et al.*, 2016 ▸). Green circles (bottom) indicate residues that coordinate the chloride ion in *Nostoc* sp. KVJ10. The blue frames indicate the putative ATP-binding sites (Liao *et al.*, 2012 ▸; Wang *et al.*, 2016 ▸).

The amino-acid sequence of nAGE10 was compared with those of other characterized AGEs from cyanobacteria, as well as those from human, pig, rat, *B. ovatus* and *P. heparinus* (Klermund *et al.*, 2013 ▸; Lee, Chien *et al.*, 2007 ▸; Maru *et al.*, 1996 ▸; Sola-Carvajal *et al.*, 2012 ▸; Tabata *et al.*, 2002 ▸; Takahashi, Takahashi, Kaneko, Ogasawara, Shindo, Saito & Kobayashi, 1999 ▸). Their sequences are presented in Fig. 6 ▸ as a multiple sequence alignment, which shows that the nAGE10 sequence is closest to that of *N. punctiforme* PCC 73102, with 96.14% sequence identity (Edgar, 2004*a* ▸,*b* ▸). AGEs are quite conserved amongst related species, with greater than 80% identity within mammalian sequences and greater than 90% in the Nostocaceae family (*Anabaena* and *Nostoc* genera). For the latter, sequences from *Anabaena* are grouped together along with that of *Nostoc* sp. PCC 7120, which is also known as *Anabaena* sp. PCC 7120 (Kaneko *et al.*, 2001 ▸). This is consistent with the currently accepted phylogenic distribution of *Nostoc* and *Anabaena* strains (Svenning *et al.*, 2005 ▸). The sequences for *P. heparinus*, *B. ovatus* and *Synechocystis* sp. stand out, which is expected considering that they do not belong to either of the two aforementioned groups.

### 3.6. Expression and purification   

The AGE from *Nostoc* sp. KVJ10 was successfully expressed as a soluble, His-tagged protein in *E. coli* BL21 Star (DE3) cells. It could be purified in one step by affinity chromatography, giving a yield of purified protein of up to 40 mg per litre of culture when grown in LB medium or 87 mg per litre of culture when using TB medium (data not shown). Gel filtration showed that nAGE10 was present as a dimer, and a melting temperature of 72.3°C was determined by DSC. The SDS–PAGE gel and the chromatograms from the gel-filtration and DSC experiments are presented in the supporting information. Together with BoAGE and SynAGE, nAGE10 is the third report of a high-yielding AGE that does not form inclusion bodies when expressed without chaperones (Datta, 1970 ▸; Klermund *et al.*, 2013 ▸, 2015 ▸; Lee, Chien *et al.*, 2007 ▸; Maru *et al.*, 1996 ▸; Sola-Carvajal *et al.*, 2012 ▸; Tabata *et al.*, 2002 ▸).

### 3.7. Verification of nAGE10 activity   

**Figure 7:**
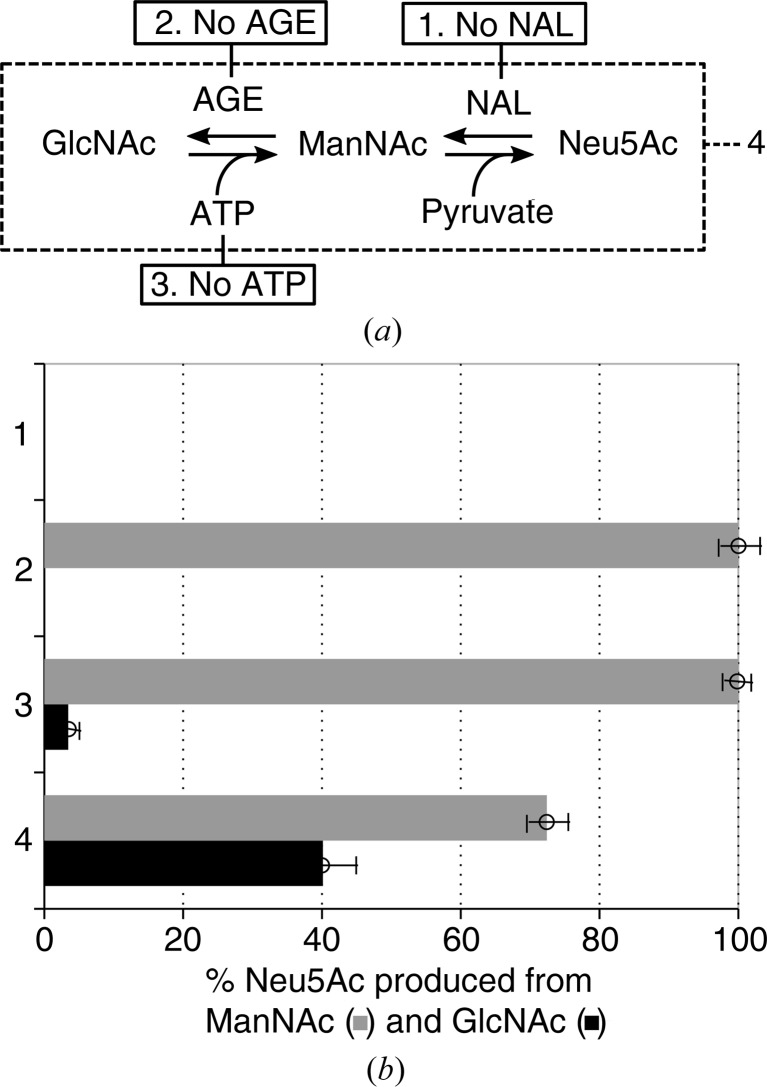
Synthesis of Neu5Ac by coupled epimerase–aldolase reactions. (*a*) Reaction scheme for the coupled reaction. Each reaction is performed using either GlcNAc or ManNAc as a substrate. 1: the reaction is performed in the absence of AsNAL and thus only the epimerization reaction occurs. 2: the reaction is performed in the absence of AGE and thus only the condensation reaction occurs. 3: the reaction is performed in the absence of ATP, affecting the epimerization reaction. 4: complete coupled reaction. (*b*) Detection of Neu5Ac by the TBA assay for reactions 1–4, using either GlcNAc (grey) or ManNAc (black) as a substrate. Production of Neu5Ac is expressed as percentage of the amount produced for the condensation reaction alone (2) using ManNAc as a substrate (grey bar). The reactions were performed at room temperature (25°C) with an incubation time of 1 h.

In order to verify that the purified nAGE10 was active, a one-pot, coupled reaction with AsNAL was performed and the production of Neu5Ac was detected by the TBA assay (Fig. 7 ▸). The reaction was performed at 25°C, according to the optimal working conditions for the NAL. The yield from the condensation reaction using ManNAc as a substrate (‘No AGE’; grey bar) was defined as 100% for the purposes of comparison with the coupled reaction. The results show that the synthesis of Neu5Ac from GlcNAc is possible and that 40% of the maximum yield could be achieved under the assay conditions. It is worth noting that different conditions can give a much higher yield (data not shown). For the condensation reaction, the presence of active AGE (*i.e.* with ATP) reduces the yield starting from ManNAc by 28%, showing that nAGE10 is active in both directions. AGE will initially speed up the formation of GlcNAc; however, when the NAL uses more of the ManNAc then the production of ManNAc by the AGE will increase. The incubation times used will determine how severe this effect is. In the absence of ATP, only a fraction of Neu5Ac could be synthetized from GlcNAc, while the condensation reaction was not affected.

## 4. Conclusion   

The crystal structure of nAGE10 presents a different dimer organization to that previously published for AGE structures, while the monomeric unit is very similar. The dimerization interface involves the front faces of each AGE monomer, and analysis revealed that this assembly is the most probable dimer association for AGEs. The front-to-front dimer organization leaves open previous hypotheses regarding the location of the ATP-binding site and raises the question of whether ATP binding affects the dimer interactions at the interface. This opens new perspectives to analyze and understand the role of ATP in the regulation of AGE activity. Another difference from the previously published AGE structures was the presence of an ethylene glycol molecule and a chloride ion within the active site of nAGE10. Comparison with the active sites of other members of the AGE superfamily, cellobiose 2-epimerases and *yihS*-encoded isomerases, suggests that the chloride ion may be a conserved element of the active site and that ethylene glycol mimics the way that substrates of AGE may bind. In addition to this, it was possible to formulate a hypothesis for the binding of the *N*-acetyl group of hexos­amines. nAGE10 can be used for the one-pot synthesis of sialic acid at 25°C when coupled with the NAL from the psychrophilic bacterium *A. salmonicida*. The insight gained from this structure offers new residues to consider for mutation studies. Together, these observations open new paths of investigation with new residues to consider, which may lead to a better understanding of AGEs.

## Supplementary Material

PDB reference: *N*-acetylglucosamine 2-epimerase, 6f04

Supplementary Figure S1.. DOI: 10.1107/S2059798318017047/jc5019sup1.pdf

## Figures and Tables

**Table 1:** Data collection and processing Values in parentheses are for the outer shell.

PDB code	6f04
Data collection	
Synchrotron-radiation source	BESSY 14.1
Detector	PILATUS 6M
Wavelength (Å)	0.9184
No. of frames	1800
Oscillation range per frame (°)	0.1
Diffraction data	
Space group	*P*4_2_2_1_2
*a*, *b*, *c* (Å)	142.423, 142.423, 52.046
Protein molecules in asymmetric unit	1
Total No. of reflections	768654 (109502)
No. of unique reflections	59456 (9464)
Resolution range (Å)	50–1.7 (1.8–1.7)
Completeness (%)	99.9 (99.7)
〈*I*/σ(*I*)〉	15.58 (1.49)
Observed *R* factor (%)	13.2 (143)
CC_1/2_	99.9 (64.3)
Refinement	
Resolution limits (Å)	48.9–1.7
No. of used reflections	59447
Data completeness (%)	99.94
Percentage of free reflections	3.5
No. of protein atoms	3214
No. of heterogen atoms	17
No. of waters	318
*R* factor/*R*_free_	0.179/0.209
Overall *B* factor from Wilson plot (Å^2^)	21.61
R.m.s.d., bonds (Å)	0.007
R.m.s.d., angles (°)	0.915
Ramachandran statistics	
Favoured	381
Allowed	5
Outliers	0

**Table:** (*a*) nAGE10.

Hydrogen bonds		vdW interactions†	
1‡	2	1	2
D48	R234	C39, D48, F52	F231
D50	R167	Y47	F231, R234, H292, P293
P108	T166	T103, L109	T166, R167
R167	D50	K107, P108	T166
R234	D48	T166	T103, K107, P108, L109
Q295	K367	R167	T103, L109
L297	Q301	F231	Y47, F52, K367
W299	L360	R234	Y47, D48
Q301	L297	Y285, Y352	L297
L360	W299	H292	Y47
K367	Q295	P293	Y47, L360, L361, N362, L363
		P294, Q296	L361
		Q295	L361, K367
		L297	Y352, L360, L361, Y285, E298
		E298	L297, E298, Q301
		W299	L360, L361
		D300	L360
		Q301	L297, Q301
		N362, L363	P293
		K367	F231, Q295

**Table:** (*b*) pAGE.

Hydrogen bonds		vdW interactions	
A1	B2	A1	B2
Y49	T304	Y49	L301, C239, T304
D50	C239	D50	C239, G238, R242
R113	P171	V97, V117	L170
P114	P171, A173	T109	G172
P171	R113	R113, P114	P171, G172
A173	P114	V115	L170, A173
C239	D50	L170	K116, V97, V115, K116
R242	Y49	P171	R113, P114
C302	T371	G172	T109, R113, P114, V115
W308	A369	A173	V115
A369	W308	G238	D50
T371	C302	C239, R242	Y49, D50
		L301	Y49
		C302	I370, T371, I372
		T308	I370
		L306	Y294, Y361, E307, M310, L312
		M310, L312, Y361	L306
		L370	C302, T304, L306, W308
		T371	C302
		I372	C302, T304
		P376	T304

**Table:** (*c*) AnaAGE.

Hydrogen bonds		vdW interactions	
A1	B2	A1	B2
D47	F230, R233	C38, F51	F230
D49	R166	D40, N361, L362	P292
T165	P107	Y46	F230, R233, H291, P292
R166	D49, T102	D47	F230, R233
F230	D47	T102, L108	R166
R233	D47	T165	T102, E106, P107, L108, V109
L296	Q300	R166	T102, L108, V109
W298	L359	F230	Y46, F51, K366
Q300	L296	R233	Y46, D47
R355	E357	Y284, Y351	L296
E357	R355	H291	Y46
L359	W298	P292	Y46, L360, N361, L362
		P293	L360
		Q294	L360, K366, W367
		L296	Y284, E297, Q300, Y351, L359, L360
		E297	L296, E297, Q300
		W298	L359, L360
		D299	L359
		Q300	L296, E297, Q300
		N353	R355
		R355	N353, R355
		L359	L296, W298, D299
		L360	P292, P293, Q294, L296, W298
		K366	F230, Q294
		W367	Q294

† Between H atoms of monomers; within 4.5 Å.

‡ Monomers. In the cases of pAGE and AnaAGE, the chain is specified.
