# Supplementary material for: The crystal structure of the N-acetylglucosamine 2-epimerase from Nostoc sp. KVJ10 reveals the true dimer
Source: Acta Crystallogr D Struct Biol. 2019 Jan 8;75(Pt 1):90–100. doi: 10.1107/S2059798318017047 (PMC6333288; doi:10.1107/S2059798318017047)
Supplement: Supplementary file 1 [file d-75-00090-sup1.pdf]

STRUCTURAL
BIOLOGY

Volume 74 (2018)

Supporting information for article:

The crystal structure of the *N*-acetylglucosamine 2-epimerase from *Nostoc* sp. KVJ10 reveals the true dimer

Marie-Josee Haglund Halsør, Ulli Rothweiler, Bjørn Altermark and Inger Lin Uttakleiv Ræder

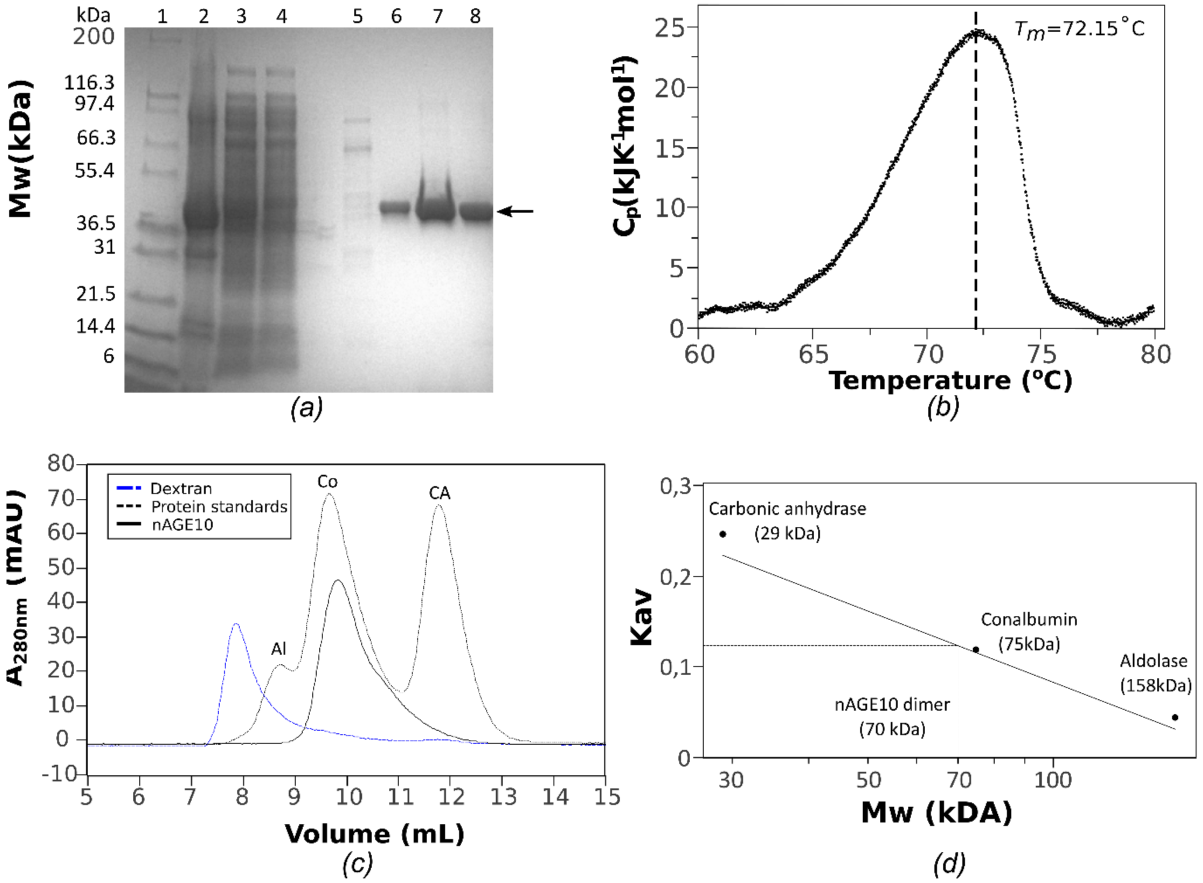

Figure S1 Purification and determination of melting temperature of nAGE10. *(a)* One-step purification of nAGE10. Lane 1: insoluble fraction. Lane 2: soluble fraction. Lane 3: Flow through. Lane 4: Washing at 5% elution buffer. Lanes 5-8: Elution fractions. The first elution fraction (lane 5) represents a peak at 25% elution buffer, while the others (lanes 6-8) encompass a peak at 60% elution buffer. A total of 37,8 mg purified nAGE10 was harvested from those. *(b)* Differential scanning calorimetry (DSC) experiment. The thermal denaturation of nAGE10 was followed from 5 to 95°C at a rate of 1°C/min. The thermogram shows the transition phase between 60 and 80°C. The melting temperature T_m is indicated by a vertical dashed line crossing the transition peak at T=72.15°C. *(c)* Size exclusion chromatography (SEC) of native nAGE10. *(d)* Standard curve for the SEC experiment.
